# Exploring proinsulin proteostasis: insights into beta cell health and diabetes

**DOI:** 10.3389/fmolb.2025.1554717

**Published:** 2025-03-05

**Authors:** Parisima Ghaffarian Zavarzadeh, Kathigna Panchal, Dylan Bishop, Elizabeth Gilbert, Mahi Trivedi, Tovaria Kee, Srivastav Ranganathan, Anoop Arunagiri

**Affiliations:** ^1^ Department of Biological Sciences, East Tennessee State University, Johnson City, TN, United States; ^2^ Max Planck Institute for the Physics of Complex Systems, Dresden, Germany

**Keywords:** proinsulin folding, trafficking, beta cells, proteostasis, insulin biosynthesis, diabetes

## Abstract

Proinsulin misfolding is central to diabetes. This review examines the cellular mechanisms regulating proinsulin proteostasis in pancreatic β-cells, encompassing genetic factors such as insulin gene mutations, and exploring the roles of endoplasmic reticulum (ER) stress and the unfolded protein response (UPR), ER redox balance, mitochondrial function, and the influence of extrinsic factors. Mutations in the INS gene, particularly those affecting cysteine residues, impair folding and disulfide bond formation, often exhibiting dominant-negative effects on the wild-type proinsulin. The importance of ER quality control mechanisms, including chaperones and oxidoreductases, in facilitating proper folding and degradation of misfolded proinsulin is emphasized. Disruptions in these systems, due to genetic mutations, ER stress, or impaired ER-to-Golgi trafficking, lead to proinsulin accumulation and β-cell dysfunction. The unfolded protein response (UPR), especially the PERK and IRE1α-XBP1 pathways, emerges as a central regulator of protein synthesis and ER stress management. The review also discusses the role of mitochondrial health, ER redox state, and extrinsic factors such as diet and medications in influencing proinsulin proteostasis. Finally, the structural insights from NMR and molecular dynamics simulations are discussedhighlighting the dynamics of misfolding and underscoring the importance of disulfide bonds. These mechanistic insights suggest innovative strategies targeting thiol/disulfide redox systems in cells to mitigate protein misfolding diseases including diabetes.

## 1 Introduction

**FIGURE 1:**
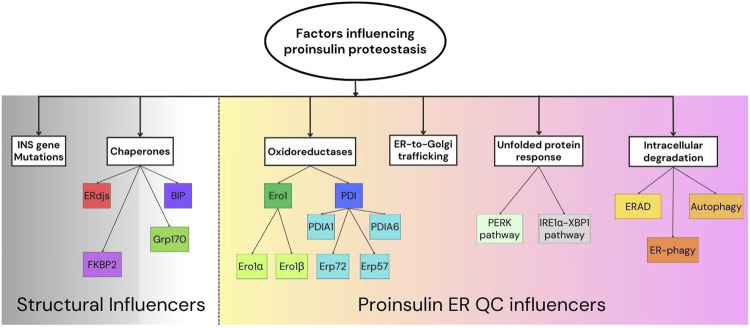
Factors influencing proinsulin proteostasis.Schematic representation of the proinsulin proteostasis “influencers” broadly divided into structural influencers (*left*) and proinsulin ER quality control (QC) mechanisms (*right*). Key components involved in proinsulin folding and ER quality control are shown.

Proinsulin, the established insulin precursor, is produced and folded within pancreatic *β*-cells’ endoplasmic reticulum (ER). The folded proinsulin trafficks from the ER to Golgi and is further processed to produce equimolar insulin and C-peptide, both of which are subsequently stored in secretory granules in the *β*-cells (Kitabchi, 1977). Proinsulin comprises 86 amino acids organized into three chains: B-chain, C-peptide, and A-chain. Critical disulfide bonds, A6-A11 (intra-chain), B7-A7, and B19-A20 (both inter-chain), ensure the native folding of proinsulin (Gupta, 2022; Haataja et al., 2016). To maintain insulin levels, pancreatic *β*-cells must also regulate proinsulin production. Proinsulin is inherently prone to misfolding, with a subset of molecules particularly susceptible to this during their synthesis. Misfolded proinsulin is generally degraded via ER-associated protein degradation (ERAD) or reticulophagy. Mutant INS-gene-induced Diabetes of Youth (MIDY), a subtype of Maturity-Onset Diabetes of the Young (MODY), caused by insulin (INS) gene mutations is historically featured proinsulin misfolding (Liu et al., 2010a; Sun et al., 2020; Haataja et al., 2021). However, proinsulin misfolding is not exclusively a consequence of genetic mutations in the INS gene. Non-mutated, wild-type (WT) proinsulin can also misfold when the ER “folding environment” becomes unfavorable, such as during ER stress. Notably, β-cell failure in type 2 diabetes (T2D), which lacks INS gene mutations, is also associated with ER stress and proinsulin misfolding. This suggests a bidirectional relationship between proinsulin misfolding and ER stress, both of which can lead to β-cell dysfunction and the development of diabetes (Laybutt et al., 2007; Arunagiri et al., 2019). Mitochondrial dysfunction and reduced cellular energy can also contribute to proinsulin misfolding (Arunagiri et al., 2024). Observation of proinsulin misfolding in prediabetic T2D rodent models (Arunagiri et al., 2019) suggests that patients with early-stage T2D and dysglycemia may exhibit proinsulin misfolding before complete *β*-cell failure. Recent research has significantly advanced our understanding of proinsulin misfolding under *β*-cell stress and in different forms of diabetes (Sun et al., 2020; Arunagiri et al., 2019; Arunagiri et al., 2024). This review will provide a comprehensive overview of the latest findings, examining genetic and cellular factors that impact proinsulin proteostasis—synthesis, folding, trafficking, and degradation, in the context of diabetes pathophysiology. Section 2 below focuses on INS gene mutations that cause proinsulin misfolding. Sections 3 through 8 then discuss the mechanisms that regulate proinsulin quality control in β-cells (Figure 1). Finally, Section 9 explores the structural basis of proinsulin misfolding.

## 2 MODY mutations

Maturity-Onset Diabetes of the Young (MODY) is a distinct group of monogenic diabetes subtypes, separate from type 1 (autoimmune) and type 2 (polygenic/environmental) diabetes (Hoffman et al., 2025; Antal, 2021). Accounting for 1%–5% of all diabetes cases, MODY is the most common form of monogenic diabetes, characterized by autosomal dominant inheritance and typically presenting before age 25 (Hoffman et al., 2025; Antal, 2021). Caused by heterozygous mutations in various genes affecting insulin secretion, MODY can exhibit a wide range of clinical presentations. Accurate diagnosis is crucial due to subtype-specific treatment responses, complication rates, and extra-pancreatic manifestations. While initially described in non-obese individuals, MODY has been reported across various racial groups (Hoffman et al., 2025). It is estimated that MODY accounts for 6.5% of antibody-negative diabetes in children (Hoffman et al., 2025). Advanced genetic testing has identified numerous MODY genes and subtypes, improving our understanding of these disorders (Hoffman et al., 2025; Antal, 2021). Some of the key MODY subtypes (Hoffman et al., 2025; Antal, 2021) influencing *β*-cell function or pancreas development include:

a) GCK MODY (MODY 2): GCK gene (7p15-p13), glucokinase. Regulates insulin secretion. Higher fasting glucose. Mild, non-progressive hyperglycemia, often asymptomatic.
b) HNF1A MODY (MODY 3): HNF1A gene (12q24.2), HNF1alpha protein. Regulates transcription in various tissues. Impaired glucose metabolism in *β*-cells. Progressive *β*-cell dysfunction. Decreased renal glycosuria threshold.
c) PDX1/IPF1-MODY (MODY 4): PDX1 gene (13q12.2), PDX1 protein. Crucial for pancreatic/*β*-cell development and insulin gene expression.
d) NEUROD1 MODY (MODY 6): NEUROD1 gene (2q32), NeuroD1 protein. Affects pancreatic/neuronal development.
e) KLF11 MODY (MODY 7): KLF11 gene (2q25), KLF11 protein. Regulates PDX1 and insulin gene expression.
f) PAX4 MODY (MODY 9): PAX4 gene (7q32), PAX4 protein. Essential for *β*-cell formation.
g) INS MODY (MODY 10): INS gene (11p15.5), preproinsulin. Mutations lead to misfolded proinsulin.
h) ABCC8 MODY (MODY 12): ABCC8 gene (11p15), SUR1 protein. Regulates insulin secretion. Associated with neonatal diabetes/hypoglycemia. Responsive to sulfonylureas.
i) KCNJ11 MODY (MODY 13): KCNJ11 gene (11p15), KIR 6.2 protein. Regulates insulin secretion. Associated with neonatal diabetes/hypoglycemia. Responsive to sulfonylureas.
j) APPL1 MODY (MODY 14): APPL1 gene (3p14.3), APPL1 protein. Involved in insulin signaling and *β*-cell survival.

While INS MODY, caused by mutations directly within the insulin gene, represents the most direct link to proinsulin proteostasis, the influence of other MODY subtypes on this crucial process deserves further scrutiny. The intricate gene regulatory and protein interaction network within *β*-cells suggests that disruptions in other MODY-related genes could indirectly impact proinsulin proteostasis. Transcription factors like HNF1A, GCK and PDX1, implicated in various MODY subtypes, orchestrate the expression of numerous genes essential for *β*-cell function, potentially altering those involved in proinsulin folding and/or processing. For instance, recent research in HNF1A-MODY has revealed impaired insulin secretion and abnormal insulin granule accumulation linked to reduced CACNA1A and SYT13 expression (Gonzalez et al., 2022), respectively. These findings raise the intriguing possibility of a connection to proinsulin trafficking or processing, echoing previously established links between ER calcium imbalances and proinsulin proteostasis. Disruptions in ER calcium homeostasis, whether due to PERK deficiency affecting proinsulin synthesis and folding (Sowers et al., 2018), or SERCA2 knockout impairing proinsulin processing (Iida et al., 2023), can significantly impact *β*-cell function. The observed calcium dysregulation in HNF1A-MODY suggests a similar pathway may be involved, though further investigation is required. Another gene, glucokinase (GCK/MODY 2), while primarily known for its role in glucose-stimulated insulin secretion, also plays a crucial role in *β*-cell mass and survival (Matschinsky and Wilson, 2019). Studies suggest that while GCK activation can have proliferative effects on *β*-cells (Wei et al., 2009), a prolonged activation can lead to *β*-cell failure due to increased ER stress (Tornovsky-Babeay et al., 2021). This delicate balance of GCK activity suggests a link to proinsulin proteostasis, as altered GCK activity could influence insulin biosynthesis and secretion, potentially impacting upstream proinsulin synthesis (and folding). Finally, studies on PDX1 (MODY 4), a transcription factor crucial for *β*-cell development and function, have revealed its importance in managing ER stress, a key player in proinsulin proteostasis. Research has shown that Pdx1 levels are maintained during ER stress through a specialized translational mechanism, allowing the *β*-cell to adapt to the stress (Templin et al., 2014; Sachdeva et al., 2009). Importantly, Pdx1 also regulates a several ER-related genes, including those involved in disulfide bond formation (e.g.,. Ero1β), protein folding (BiP), and the UPR itself (Sachdeva et al., 2009). Pdx1 deficiency leads to increased ER stress, highlighting its critical role in maintaining ER homeostasis. Given the strong links recently established between ER homeostasis and proinsulin folding and trafficking (Arunagiri et al., 2019; Arunagiri et al., 2024; Rohli et al., 2024), it is highly likely that PDX1 plays a significant role in proinsulin proteostasis. Notwithstanding the foregoing, the precise mechanisms and extent of these indirect effects from various MODY subtypes remain to be fully elucidated. While different MODY subtypes could potentially influence proinsulin proteostasis (with limited published data available), extensive literature supports the fact that INS MODY (MODY 10) most directly relates to proinsulin folding and trafficking because mutations in the *INS* gene can result in proinsulin structural defects and cause misfolding. The following subsection is dedicated to exploring this critical link.

### 2.1 MIDY (MODY 10)/INS gene mutations

Mutations in the INS gene can cause Mutant INS-gene-induced Diabetes of Youth (MIDY) by disrupting the proper folding of proinsulin. The misfolded mutant proinsulins containing free cysteine residues can oligomerize via intermolecular disulfide bridges (Sun et al., 2020; Haataja et al., 2021). Besides that they form disulfide bonds with WT proinsulin, resulting in the formation of hetero-oligomers within the *β*-cells (Sun et al., 2020). Regardless of whether the mutation introduces or eliminates a free cysteine residue, the mutated proinsulin tends to sequester WT proinsulin through intermolecular bonding. This dominant-negative effect of the mutant on WT proinsulin occludes ER-to-Golgi export of WT proinsulin, potentially impairing insulin production in the *β*-cell. This interplay between mutant and WT proinsulin underlies the pathogenesis of MIDY (Liu et al., 2010a).

**FIGURE 2:**
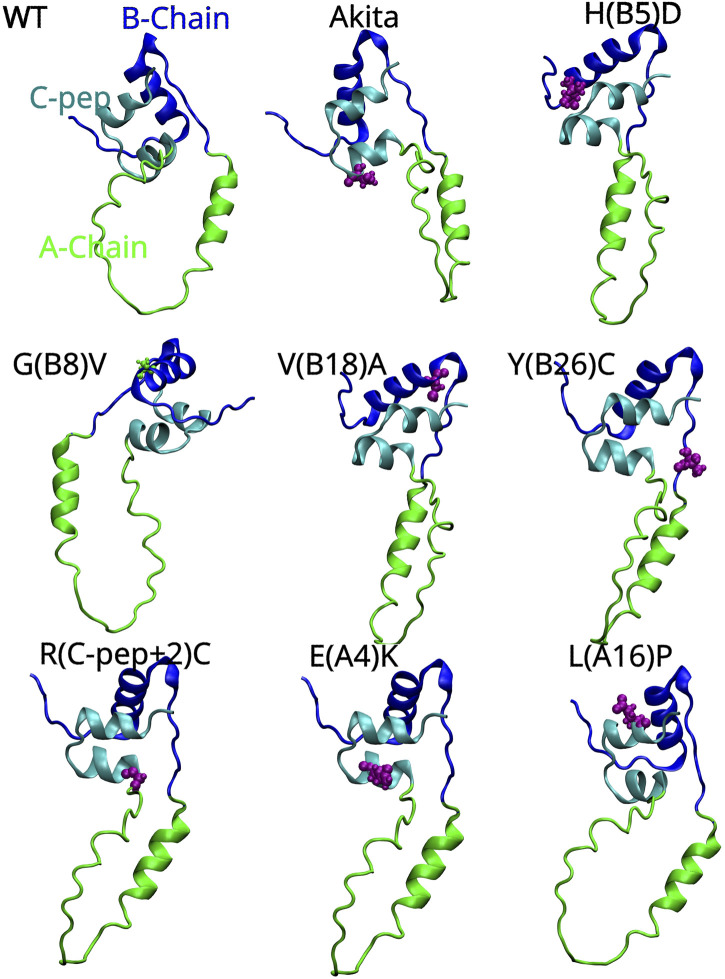
AlphaFold-predicted 3D structures of human proinsulin with the A-Chain, B-Chain and C-peptide represented as green, blue and cyan colored ribbons respectively. The locations of the *Akita* (C (A7)Y) and MIDY mutations are shown in pink color and van der Waals spheres showing their 3D location with respect to the three key regions of proinsulin. The proinsulin mutant nomenclature is as follows: H(B5)D means Histidine, which is the fifth amino acid on the B-chain, is replaced with Aspartic acid.

MIDY can be categorized based on mutation type, as not all INS mutations lead to the disease. Several missense mutations (Figure 2) can affect proinsulin folding and cause MIDY (Liu et al., 2010a; Haataja et al., 2021). These mutations disrupt critical disulfide bonds necessary for proper protein folding. While different mutations have varying effects on proinsulin folding, alterations in highly conserved residues are more strongly associated with the MIDY phenotype (Liu et al., 2010b). Alternatively, MIDY may be categorized based on the impact of the mutation on proinsulin biosynthesis and folding. Although INS gene mutations can impair proinsulin folding, their severity and specific effects vary. For instance, the C43G mutation disrupts the formation of the B19-A20 disulfide bond, one of the earliest bonds formed in native proinsulin, significantly affecting its folding and secretion. In contrast, the G32R and Y108C mutations produce proinsulin that can enter the secretory pathway but is functionally compromised. The severity of the mutations can indirectly be assessed by measuring the amount of intracellular and extracellular (secreted) insulin or C-peptide (Park et al., 2010).

Mutations within the proinsulin sequence, particularly on the B- and A-chains, can disrupt folding in MIDY. This is especially true for mutations in conserved residues like HisB5 and GlyB8, which are critical for proper folding of proinsulin. The limited potential for compensatory mutations due to the conservative nature of the proinsulin sequence further exacerbates the deleterious effects of misfolding, leading to clinical diabetes (Weiss, 2013). A prime example is Tyrosine-B16 (Tyr-B16), a crucial residue for normal proinsulin dimerization. While mutations like Y(B16)D or Y(B16)A primarily affect interactions and lead to dimerization without directly impacting folding or secretion, the Y(B16)P mutation specifically induces misfolding. Additionally, Tyr-B16 can contribute to the dominant-negative effect by facilitating interactions between WT and misfolded proinsulin. This effect is particularly pronounced in the C(A7)Y *Akita* mutation, which introduces a free cysteine residue and promotes the formation of disulfide-linked oligomers that impair the secretion of WT proinsulin. Interestingly, substituting Tyr-B16 with Ala, Asp, or Pro can mitigate this dominant-negative effect by preventing these aberrant interactions (Sun et al., 2020). Mutations affecting non-cysteine residues can also disrupt proinsulin folding and secretion. For instance, the G(B23)V mutation hinders the proper assembly of the A and B chains, preventing the formation of disulfide bonds. This finding suggests that mutations outside of cysteine residues can still impair proinsulin structure, and thereby the structure of insulin, and possibly its function too (Liu et al., 2010a). Several non-cysteine mutations have been shown to block proinsulin secretion from the ER. Some of these mutations, such as those affecting the Cys (A6)–Cys (A11) bond, disrupt proper folding and trafficking. However, the severity of these mutations varies. While some, like V(B18)A and E(A4)K, can partially escape quality control and be secreted, others, such as L(A16)P and H(B5)D, have more severe effects and are largely retained in the ER (Haataja et al., 2021), indicating that the amino acid substitution and the position of substitution on proinsulin (primary sequence) plays a pivotal role in determining the magnitude of misfolding and proteotoxicity. Different mutations can impair proinsulin folding, trafficking, and ER stress response, leading to varying degrees of diabetes severity. While some mutations, such as G(B20)R and P(B28)L, have milder effects and are associated with the disease, others, like C(A7)Y, have more severe consequences. The G(B20)R mutation interestingly leads to increased binding affinity of proinsulin to the insulin receptor but disrupts protein folding and trafficking, ultimately leading to reduced insulin secretion and contributing to the development of diabetes (Wang et al., 2020). It was recently realized that regardless of the mutation location within the proinsulin sequence, misfolding often involves the formation of disulfide-linked oligomers through interactions with free thiol groups on the proinsulin molecule (Haataja et al., 2021).

These studies overall emphasize the importance of understanding the mechanisms underlying proinsulin misfolding and the impact of specific mutations on proinsulin structure, insulin production and secretion. By elucidating these mechanisms, researchers may be able to develop novel therapy for treating diabetes caused by INS gene mutations (Haataja et al., 2021). The proposed strategies along these lines include i) a combination of CRISPR/Cas9 and stem technology to replace deleterious mutations implicated in MIDY (Balboa et al., 2018) or ii) devising approaches to target free thiols in proinsulin to mitigate intermolecular disulfide pairings (Haataja et al., 2021).

Proinsulin folding and trafficking, in addition to being affected by INS gene mutations, are also heavily influenced by the ER microenvironment. The ER’s resident proteins and processes directly or indirectly impact proinsulin folding. After a brief overview of ER stress and the unfolded protein response, the following sections will delve into these influential factors.

## 3 ER stress and Unfolded Protein Response (UPR)

The ER is a vital organelle responsible for numerous cellular functions, including protein folding, lipid synthesis, and calcium regulation (Lodish et al., 2021). ER stress arises when these functions are disrupted, often due to an accumulation of misfolded or unfolded proteins in the ER lumen. This protein buildup, known as proteotoxicity, overwhelms the ER’s capacity to properly fold proteins (Oakes and Papa, 2015). While proteotoxicity is a common cause, other stressors like disruptions in lipid metabolism, glucose regulation, or calcium homeostasis can also trigger ER stress. Essentially, anything that throws the delicate balance of the ER’s functions off can lead to stress. The cell’s primary response to ER stress is the Unfolded Protein Response (UPR) (Ron and Walter, 2007). The UPR is a complex signaling pathway with the goal of restoring ER homeostasis. It has three main branches, each activated by distinct transmembrane proteins in the ER membrane:

i. IRE1α: When activated by unfolded proteins (indirectly via BiP, as discussed below), IRE1α splices XBP1 mRNA, which then encodes a transcription factor that upregulates genes involved in protein folding and ER-associated degradation (ERAD) (Ron and Walter, 2007; Hetz et al., 2020).
ii. PERK: PERK phosphorylates eIF2α, a protein involved in translation initiation. This phosphorylation has a dual effect: it reduces overall protein synthesis, lessening the burden on the ER, and it selectively increases the translation of certain mRNAs, including ATF4, which encodes a transcription factor that induces genes involved in protein folding, autophagy and antioxidative response (Hetz et al., 2020).
iii. ATF6: ATF6 is a transmembrane protein that, when activated by ER stress, translocates to the Golgi apparatus. In the Golgi, it is cleaved, releasing the active ATF6 fragment, which then travels to the nucleus to activate the transcription of genes involved in protein folding, ER/Golgi biogenesis and ERAD (Hetz et al., 2020).

The role of BiP in the UPR. BiP (Binding immunoglobulin protein), also known as GRP78, is a major ER chaperone. It belongs to the Hsp70 family of proteins and plays a crucial role in protein quality control within the ER, mostly by activating the three UPR branches, as highlighted below.

(a) BiP acts as a key regulator of the IRE1α branch of the UPR. The two models describe how BiP interacts with IRE1α: (1) The “BiP competition” model (Adams et al., 2019): In the absence of ER stress, BiP is bound to IRE1α, keeping it inactive. When unfolded proteins accumulate in the ER, BiP preferentially binds to these unfolded proteins, effectively “competing” for BiP. This dissociation of BiP from IRE1α allows IRE1α to become activated and initiate the UPR signaling cascade, and (2) A second model suggests that BiP directly senses the presence of unfolded proteins and then transmits this information to IRE1α, leading to its activation. Essentially, BiP acts as a sensor of ER stress and relays this information to IRE1α (Carrara et al., 2015; Kopp et al., 2018). Regardless of the precise mechanism, BiP’s interaction with IRE1α is essential for initiating the UPR in response to ER stress. It acts as a molecular switch, linking the presence of unfolded proteins to the activation of the UPR pathway.
(b) PERK, like IRE1, relies on the chaperone protein BiP for its activation (Carrara et al., 2015). BiP normally binds to the luminal domains of both IRE1 and PERK, keeping them inactive. However, when unfolded proteins accumulate in the ER lumen, BiP’s affinity for these unfolded proteins surpasses its affinity for IRE1 or PERK. BiP dissociates from PERK (and IRE1) and binds to the unfolded proteins. This release of BiP from PERK allows PERK molecules to oligomerize and become trans-phosphorylated, which is the crucial step that activates PERK (Cui et al., 2011). BiP’s binding to PERK is thought to sterically hinder the oligomerization process, preventing PERK from coming together to activate itself (Bertolotti et al., 2000). Once PERK is activated through this BiP-mediated release and oligomerization, it proceeds to phosphorylate eIF2α, leading to the downstream effects on translation (Harding et al., 1999).
(c) BiP regulates of ATF6 activation. BiP binds to ATF6’s luminal domain, utilizing its peptide-binding domain in a manner similar to its interaction with unfolded proteins, forming a stable complex. This binding serves to inhibit ATF6 activation by preventing its transport to the Golgi apparatus, where it undergoes proteolytic cleavage to become active (Shen et al., 2005). During ER stress, BiP’s higher affinity for accumulating unfolded proteins leads to its release from ATF6. This release is not a passive consequence of competition but is actively triggered by specific sequences within ATF6. Once freed from BiP, ATF6 can then translocate to the Golgi, where it is cleaved and becomes transcriptionally active (Shen et al., 2005). Therefore, BiP functions as a master regulator of the ATF6 branch of the UPR, maintaining ATF6 in an inactive state within the ER under non-stressful conditions and releasing it only when ER stress signals the necessity for UPR activation to restore cellular homeostasis.

In addition to BiP, several other ER chaperones contribute to quality control within the ER. Given this review’s focus on the β-cell ER and proinsulin proteostasis, the following subsection will introduce and describe the roles of key chaperones found in the β-cell ER, beginning with BiP. The individual branches of the unfolded protein response will then be discussed. Finally, we will explore the connection between proinsulin synthesis and the UPR in β-cells, followed by a separate discussion of the relationship between calcium homeostasis and the UPR in these cells.

### 3.1 ER chaperones

#### 3.1.1 BiP

Chaperones are essential proteins that assist in proper protein folding within the cell. Among these, BiP, a member of the HSP70 family residing in the ER, plays a pivotal role. BiP interacts with various co-chaperone families, such as the DnaJ/HSP40 family, which stimulate its ATPase activity (Pobre et al., 2019). Extensive research has demonstrated BiP’s function in facilitating proper proinsulin folding. A study employed nonreducing Tris-Tricine-urea-SDS-PAGE, a technique sensitive to disulfide mispairing, to investigate the folding differences between wild-type (WT) and mutant proinsulin (Liu et al., 2005). To track proinsulin, insulin, and conversion intermediates, a 5 and 30-min chase medium was analyzed using immunoprecipitation. This study identified two bands migrating slower than native proinsulin. One of these bands co-precipitated with BiP upon anti-BiP immunoprecipitation. Notably, these slow-migrating bands failed to convert to insulin during the chase. Their association with BiP suggests abnormal conformations, leading to their recognition by the ER quality control system. This system could either refold misfolded proinsulin oligomers or facilitate their degradation (Liu et al., 2005). Another early study on the *Akita* mouse model revealed decreased secretory granules and insulin levels, which could be attributed to lower WT proinsulin levels in the β-cell. Furthermore, the ER lumen was enlarged to accommodate the abundant misfolded proinsulin. The presence of high molecular weight proinsulin in the ER coincided with BiP overexpression likely for the degradation of these misfolded proinsulin molecules. In other words, the ER accumulation of proinsulin is possibly mitigated by BiP (Wang et al., 1999). Finally, a recent study highlighted BiP’s role in proinsulin folding by treating a rodent *β*-cell line with the bacterial SubAB protease, which targets and cleaves BiP’s “linker” region (Arunagiri et al., 2019). This treatment resulted in large, disulfide-linked proinsulin complexes retained within the ER, as confirmed by immunoblotting and immunostaining. These findings strongly suggest that BiP plays a crucial role in either refolding or degrading misfolded WT proinsulin, thereby maintaining ER homeostasis. While elevated levels of BiP co-chaperone, p58ipk, have been observed in diabetic rodent models (Laybutt et al., 2007; Arunagiri et al., 2019), it is not known whether BiP protein levels rise. Further comprehensive studies are required to clarify the precise timing of the changes in ER chaperone levels during disease progression.

#### 3.1.2 ER DnaJ-like (ERdj) family members

These ERdjs function as co-chaperones for BiP. One specific ERdj, ERdj3, is found ubiquitously throughout the body, with highest expression in secretory tissues (Shen and Hendershot, 2005). This is one of the mammalian ER DnaJ homologous that stimulates the BiP ATPase activity. Studies reveal high expression levels of ERdj3 in the pancreas, suggesting it may specifically interact with proinsulin during its production. Notably, co-immunoprecipitation experiments using an *Akita* mouse *β*-cell line harboring the proinsulin mutation (C (A7)Y, described earlier) showed that ERdj3 associates with both wild-type and mutated proinsulin (Gorasia et al., 2016). Another ERdj linked to insulin biosynthesis is ERdj4. Inactivation of the ERdj4 gene in mice resulted in chronic ER stress and *β*-cell loss, mimicking diabetic conditions (Fritz et al., 2014). Overall, understanding the function of ERdjs, particularly ERdj3 and ERdj4, holds promise for developing strategies to prevent *β*-cell dysfunction and improve glucose control in metabolic disorders like type 2 diabetes.

#### 3.1.3 Grp170

Grp170, a chaperone with unique properties, contributes to proinsulin quality control. While Grp170 can interact with BiP and influence its function, it appears to have independent roles in proinsulin handling, especially in misfolded *Akita* proinsulin. Grp170 specifically targets high-molecular-weight aggregates of *Akita* proinsulin for degradation (Cunningham et al., 2017). (Cunningham et al., 2019) reported that the ER employs two proteins, Grp170 and Reticulon 3 (RTN3), to address proinsulin aggregation. Grp170 prevents aggregation, while RTN3 facilitates the removal of aggregates via ER-phagy (Cunningham et al., 2019). These combined efforts reduce aggregate burden, restore WT proinsulin transport, and alleviate ER stress. Grp170 preferentially binds to aggregation-prone amino acid sequences, which may be exposed on the surface of *Akita* proinsulin aggregates. This interaction could promote the formation of smaller, oligomeric species that are more amenable to ERAD or refolding. The exact mechanism by which Grp170 targets *Akita* aggregates remains unclear. Understanding this interaction is crucial for elucidating the role of Grp170 in mitigating ER stress and preventing the accumulation of proinsulin aggregates in MIDY.

#### 3.1.4 *FK506-Binding* protein 2 (FKBP2)

FKBP2, a proline isomerase residing in the ER, plays a crucial role in proinsulin folding (Hoefner et al., 2023). It interacts with both proinsulin and the Grp94 chaperone, suggesting a chaperone-like function. This makes it a potential biomarker for early diabetes diagnosis and a target for improving proinsulin folding. Studies carried out by (Hoefner et al., 2023) using FKBP2 knockout (KO) models highlight its importance. In the FKBP2 KO, a significant increase in misfolded, poorly soluble proinsulin, alongside a decrease in overall ER proinsulin levels was observed, implying that misfolded proinsulin might be degraded promptly rather than aggregating. Interestingly, FKBP2 KO also triggered apoptosis in *β*-cells without inducing ER stress responses. FKBP2 appears to preferentially bind to unfolded, reduced proinsulin–specifically before disulfide bond formation–emphasizing its critical role in this early folding step. This aligns with the observation in FKBP2 KO models, where misfolded proinsulin formed disulfide-linked dimers and multimers (Hoefner et al., 2023). Paradoxically, FKBP2 was found to be overexpressed in *β*-cells of T2D patients. It was found that FKBP2 isomerizes a specific proline residue (P28) at position 28 of the proinsulin B-chain, and this isomerization, particularly converting cis-proline to the trans conformation, is crucial for releasing proinsulin from misfolded states. This is supported by proinsulin misfolding increases when FKBP2 is deficient, as evidenced by CRISPR/Cas9 KO of FKBP2 in INS-1E cells, which reduced intracellular proinsulin and insulin levels. It is clear from these findings that there is a complex relationship between FKBP2 and proinsulin folding in *β*-cells, with potential implications for both diagnosis and treatment of diabetes (Hoefner et al., 2023).

### 3.2 Role of the individual UPR branches in proinsulin proteostasis

The *β*-cell UPR arms play a crucial role in maintaining proinsulin folding and trafficking. Here, we explore the specific contributions of the PERK, IRE1α–XBP1 and ATF6 pathways, and highlight the influence of UPR in proinsulin synthesis and ER calcium homeostasis.

#### 3.2.1 PERK

**FIGURE 3:**
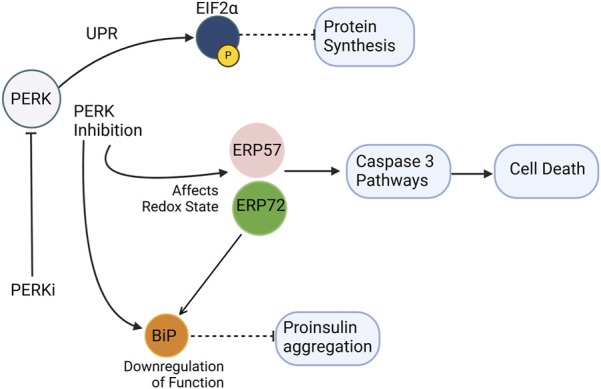
PERK inhibition disrupts ER homeostasis and promotes proinsulin misfolding. PERKi (PERK inhibitor) impairs translational attenuation, alters the ER redox environment, and downregulates chaperone function. Specifically, PERKi prevents the phosphorylation of eIF2α, leading to increased protein synthesis and exacerbating ER stress. Additionally, PERKi can affect the redox state of ER oxidoreductases like Erp72 and Erp57, potentially reducing the activity of the ER chaperone BiP. These combined effects result in the accumulation of misfolded proinsulin, contributing to ER dyshomeostasis in β-cells. Separately, Erp57 suppression results in elevated cleaved Caspase-3, indicating increased apoptosis. *Dashed inhibitory arrows indicated weakened effects.*

The PERK pathway plays a central role in maintaining proinsulin production and folding within the ER (Figure 3). The PERK/eIF2α pathway involves the phosphorylation of EIF2α by PERK kinase activity, which is crucial for protein translation in cells (Shi et al., 1998). Dysregulated eIF2α phosphorylation has been implicated in monogenic and polygenic diabetes, as β-cells in these conditions exhibit markers of unresolved ER stress (Cnop et al., 2017). The phosphorylation of eIF2α slows global protein synthesis during ER stress, allowing for the selective translation of stress-response proteins like ATF4. However, excessive or insufficient phosphorylation of eIF2α can impair β-cell survival, highlighting the delicate balance required for proper UPR signaling. A selective small molecule inhibitor, PERKi, was shown to acutely inhibit PERK’s ability to phosphorylate eIF2a thereby having immediate effects on protein synthesis in normal *β*-cells. This acute inhibition of PERK was suggested to result in the deregulation of protein synthesis causing misfolded proinsulin to accumulate (Harding et al., 2012). However, the detection of misfolded proinsulin by biochemical means revealed that the aggregation of proinsulin did not occur immediately in *β*-cells treated with PERKi, but took at least 10–12 h (Arunagiri et al., 2019). Does this mean that the PERKi-mediated ER build-up of proinsulin is independent of the protein synthesis defects resulting from PERK inhibition? A separate study by Sowers et al., 2018 highlighted that PERK could modulate the function of the ER chaperone BiP by regulating its glucose-dependent activity (Sowers et al., 2018). Disrupting this balance through PERK inhibition negatively affects BiP function, and both upregulation and downregulation of BiP can trigger proinsulin aggregation. Interestingly, while overexpression of ERp72 can rescue proinsulin from aggregation, its role seems less critical compared to BiP (Sowers et al., 2018). PERK-deficient mice models exhibit diabetic characteristics and increased vulnerability to proinsulin misfolding. This suggests that PERK dysfunction initiates ER stress, which in turn contributes to proinsulin misfolding, and the observed proinsulin aggregation might be a consequence of the inhibited pathway leading to an accumulation of abundant newly synthesized proinsulin over time in the ER (Sowers et al., 2018).

#### 3.2.2 IRE1α–XBP1 pathway

**FIGURE 4:**
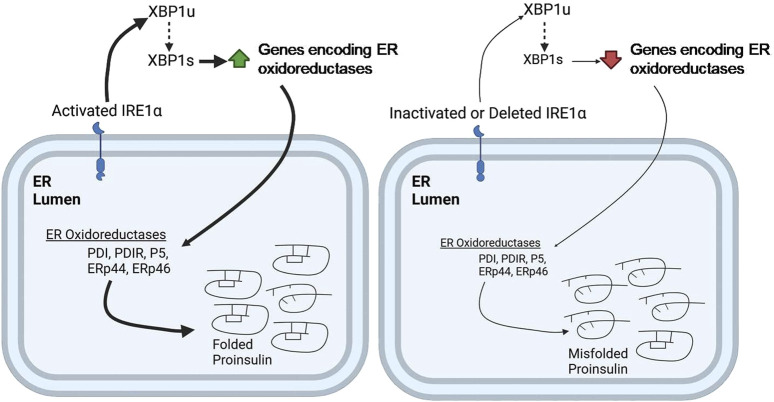
IRE1α deficiency impairs ER oxidative folding of proinsulin. IRE1α activation in pancreatic β-cells promotes XBP1 splicing, leading to increased expression of the indicated ER oxidoreductases, which enhances proinsulin folding (*Left*). In contrast, IRE1α inactivation or knockdown impairs XBP1 splicing, leading to decreased expression of ER oxidoreductases, which results in defective oxidative protein folding, particularly of proinsulin, in the β-cell ER (*right*). *Thickness of the arrows signifies the efficiency or magnitude of the processes.*

UPR triggers IRE1α–XBP1 pathway and the activation of IRE1α′s RNase domain by ER stress causes the cleaving of unspliced XBP1 (XBP1u) mRNA (X-box-binding protein 1). The spliced form, XBP1s, then acts as a transcriptional activator that upregulates specific gene encoding ER chaperones, ERAD proteins, and lipid synthesis enzymes. Tsuchiya et al., 2018 suggested that the IRE1α–XBP1pathway controls the expression of several protein disulfide isomerases (PDIs), including PDI, PDIR, P5, ERp44, and ERp46, which are essential for the oxidative folding of proinsulin (Tsuchiya et al., 2018). It was shown that inactivation of the IRE1α-XBP1 pathway leads to decreased expression of these PDIs, resulting in impaired proinsulin folding and reduced insulin production. Conversely, overexpression of these PDIs can restore insulin secretion. These findings highlight the importance of the IRE1α-XBP1 pathway in regulating proinsulin folding and the essential role of PDIs in this process (Tsuchiya et al., 2018) (Figure 4). Additionally, the deletion of Xbp1 in mouse β-cells primarily affects the expression of mRNA encoding insulin, impacting its processing and secretion (Tsuchiya et al., 2018). This further underscores the complex role of the IRE1α-XBP1 pathway in maintaining β-cell function and insulin production.

#### 3.2.3 ATF6 pathway

ATF6, a key component of the UPR and the third major arm alongside PERK and IRE1, plays a critical role in maintaining pancreatic β-cell health. Studies reveal that key UPR components, including ATF6 and sXBP1, are progressively downregulated in β-cells of both mouse models and human T1D patients, implicating UPR dysfunction in the disease process (Engin et al., 2013). This dysfunction is particularly important in the β-cells because proper folding and trafficking of proinsulin depends heavily on ER homeostasis. The accumulation of misfolded proinsulin can trigger ER stress, potentially exacerbating UPR dysfunction. Conversely, a compromised UPR, particularly the ATF6 pathway, may impair the ER’s capacity to correctly handle proinsulin, creating a vicious cycle. The chemical chaperone TUDCA shows promise in mitigating this dysfunction, preventing T1D development in mouse models by reducing disease incidence, preserving insulin production, and mitigating immune cell infiltration (insulitis) without broadly suppressing the immune system (Engin et al., 2013). Interestingly, TUDCA’s benefits depend on ATF6; in β-cell specific ATF6mice, TUDCA’s protective effects are abolished, highlighting ATF6’s crucial role in TUDCA’s mechanism (Engin et al., 2013). TUDCA appears to preserve ATF6 expression and function, essential for β-cell survival under ER stress and immune attack. Furthermore, overexpressing ATF6 in β-cells provides partial protection against ER stress and cytokine-induced death, reinforcing its importance in β-cell resilience (Engin et al., 2013). Beyond its role in T1D, ATF6 also plays a key role in β-cell proliferation (Sharma et al., 2015). Mild ER stress, activating the UPR, promotes glucose-dependent β-cell proliferation, and this effect is specifically driven by ATF6. Inhibiting ATF6 reduces proliferation, while overexpressing ATF6 increases β-cell division in both mouse and human β-cells (Sharma et al., 2015). Moreover, ATF6 acts as a central regulator within the broader UPR, controlling the transcriptional output of multiple UPR pathways, including XBP1, and playing a vital role in managing ER stress (Sharma et al., 2020). ATF6 is not only essential for the induction of its own target genes, but also required for the expression of genes targeted by the XBP1 pathway. This means that even though XBP1 activation (Xbp1 splicing) can proceed independently of ATF6, the subsequent upregulation of XBP1 target genes—crucial for alleviating ER stress—is heavily dependent on ATF6’s presence (Sharma et al., 2020). XBP1s regulates the expression of ER chaperones and protein disulfide isomerases (PDIs) (see section 3.2.2 or Figure 4), both of which, are essential for proper proinsulin foldingin β-cells. Given proinsulin folding relies on this coordinated UPR activity, including the ATF6 and IRE1α-XBP1 pathways, targeting the UPR, and specifically these pathways, offers a novel therapeutic avenue for diabetes.

#### 3.2.4 Proinsulin synthesis and UPR

Glucose elevation stimulates proinsulin translation in *β*-cells to meet increased insulin demand. However, in diabetic conditions, high proinsulin levels are observed despite diminished insulin secretion, suggesting a compensatory upregulation of proinsulin synthesis. This phenomenon, characterized by increased proinsulin production alongside reduced insulin secretion, has been documented in both human and rodent models (Ward et al., 1987; Leahy, 1993; Uchizono et al., 2007; Halban et al., 1983). The surge in proinsulin synthesis can overwhelm the ER’s folding capacity, leading to proinsulin misfolding and ultimately inducing the UPR. One of the key UPR sensors, PERK, regulates protein synthesis in cells. From a proinsulin synthesis perspective, the mechanism by which PERK inhibition leads to proinsulin misfolding/aggregation remains unclear, however as discussed in Section 6.1, aggregation likely arises from either excessive proinsulin production, impaired folding machinery, or a combination of both (Sowers et al., 2018). Recent studies have identified OSGEP as a key regulator of proinsulin translation and ER stress homeostasis in β-cells. OSGEP deletion in mice leads to impaired proinsulin translation and increased ER stress, resulting in glucose intolerance and hyperglycemia. These findings suggest that OSGEP may play a role in maintaining proinsulin proteostasis and β-cell function, particularly under conditions of high glucose and insulin demand (Liu et al., 2024).

#### 3.2.5 *ER* calcium homeostasis and UPR

ER calcium homeostasis is crucial for proper *β*-cell function and insulin production. SERCA2 (Sarco/endoplasmic reticulum calcium ATPase 2), an ER-localized calcium pump, plays a key role in maintaining ER calcium stores. (Iida et al., 2023) investigated the role of SERCA2 in pancreatic *β*-cell function and insulin production, finding that *β*-cell-specific SERCA2 knockout mice and human islets exposed to high glucose and palmitate exhibited impaired calcium signaling, altered proinsulin processing, and reduced insulin secretion (Iida et al., 2023). This highlights the importance of ER calcium homeostasis in maintaining *β*-cell function and insulin production. This study raises a question of whether ER calcium imbalance directly affects proinsulin folding. Interestingly, PERK inhibition, which perturbs proinsulin synthesis and folding (discussed earlier), was also found to interfere with calcium fluxes in the cells (Sowers et al., 2018; Wang et al., 2013), indicating a potential connection between UPR, calcium balance, and proinsulin proteostasis in *β*-cells. A good example for this is the close connection between ER calcium levels, BiP, and proinsulin folding, with BiP acting as a central component in this relationship. The ER carefully controls its calcium levels through a balance of calcium entry, removal, and buffering. In addition to the cell signaling role, calcium regulation very likely supports ER’s protein-folding machinery through ER-resident chaperones like BiP. While some studies explore its potential contribution to ER calcium storage (Lievremont et al., 1997), its well-established function is as a chaperone, assisting in the correct folding of newly synthesized proteins, including proinsulin. As discussed in section 3.1.1, BiP helps prevent proinsulin from misfolding and clumping together (Arunagiri et al., 2019). Correct calcium levels are needed for BiP to work efficiently (Preissler et al., 2020). Additionally, BiP itself can be targeted by toxins like SubAB (see section 3.1.1). When SubAB cleaves BiP, proinsulin misfolding occurs, independent of calcium levels. To sum up, maintaining correct calcium levels is important for BiP function, and BiP is essential for proper proinsulin folding. Problems at any point in this system—whether through direct calcium imbalance or BiP destruction—can lead to ER stress, activation of the UPR, and impaired proinsulin handling in the *β*-cells. While the calcium-dependent ER chaperone calreticulin (Michalak, 2024) might play a role in proinsulin proteostasis in *β*-cells, its precise function along these lines remains unknown and requires further investigation.

Given ER stress and UPR activation are so closely tied to calcium homeostasis, it is crucial to understand the dynamics of UPR activation in response to varying degrees of calcium depletion. A recent study by Pontisso et al. (2024) has shed light on this relationship, examining the early activation of UPR pathways in response to moderate ER calcium depletion. It was demonstrated that IRE1 demonstrates the fastest and most sensitive response, reaching peak phosphorylation rapidly, while PERK activation is slower and less sensitive, increasing gradually over time (Pontisso et al., 2024). ATF6 activation mirrors PERK’s pattern but exhibits bimodal dose sensitivity. Importantly, the study also found that IRE1 and PERK activation is reversible upon restoration of ER calcium levels, with phosphorylation decreasing and returning to basal levels when the calcium depletion inducer is removed (Pontisso et al., 2024). However, computational modeling, while predicting this reversibility, underestimated the speed of deactivation, suggesting the involvement of additional regulatory mechanisms, such as phosphatase activity, in the rapid recovery of IRE1 and PERK. Mechanistically, the study proposes that differences in the early steps following BiP unbinding, specifically variations in BiP affinity and oligomerization states, contribute to the distinct activation kinetics observed for each UPR branch, with IRE1 exhibiting a lower BiP affinity and autophosphorylating in larger oligomers compared to PERK (Pontisso et al., 2024).

From a *β*-cell perspective, these dynamics are critical. The ability of the UPR to sense and respond to even moderate calcium fluctuations allows *β*-cells to fine-tune their protein folding machinery and maintain proinsulin homeostasis. However, excessive calcium dysregulation, such as that seen in type 2 diabetes (Becerra-Tomas et al., 2014), can lead to persistent UPR activation, overwhelming the *β*-cell’s capacity to cope with ER stress. Such a stress coupled with the observed discrepancies in UPR deactivation kinetics, can disrupt proinsulin folding and/or processing and impair insulin production, and ultimately contribute to *β*-cell failure.

## 4 Oxidoreductases and related proteins

### 4.1 Protein disulfide isomerases (PDI)

**FIGURE 5:**
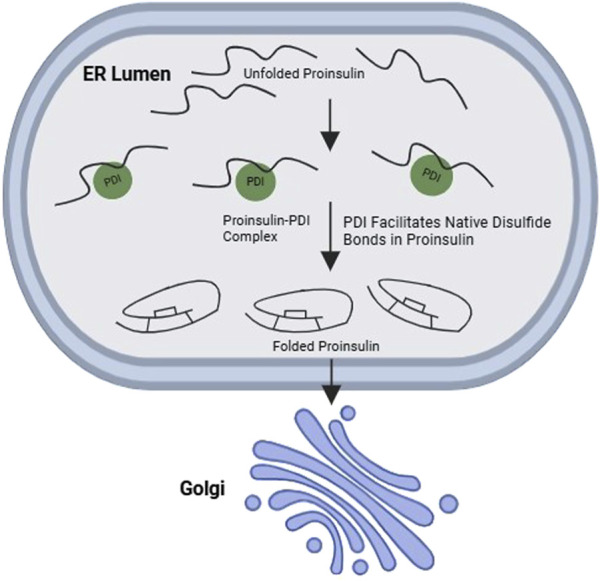
Interaction between PDI and wild-type proinsulin promotes oxidative folding of proinsulin. PDI, a major ER oxidoreductase, promotes the native disulfide bond formation in proinsulin, thereby facilitating its ER-to-Golgi transport in in β-cells.

PDI, a member of the ER oxidoreductase family, plays a critical role in protein folding within the ER. PDI itself is a complex enzyme system, with isoforms like PDIA1, PDIA6, Erp72, and Erp57 contributing to its function (Rajpal et al., 2012). Highly expressed in pancreatic *β*-cells, PDI directly influences disulfide bond formation in target proteins like proinsulin (Figure 5), making it crucial for proper insulin production. PDI participates in protein folding by acting as a “redox-active chaperone”, both preventing protein aggregation and catalyzing the formation, breakage, and rearrangement of disulfide bonds, which are vital for achieving the final protein structure. PDI is essential for efficient proinsulin refolding, acting as both a chaperone and isomerase (Winter et al., 2002). PDI’s chaperone activity is crucial in the first few seconds to prevent proinsulin aggregation, while its isomerase activity, though most effective early, can still improve folding later. Aggregation occurs rapidly at the start of refolding, and PDI’s chaperone function directly suppresses it, enabling the isomerase activity to correct disulfide bonds (Winter et al., 2002). Inhibiting PDI’s chaperone function with genistein increases aggregation and reduces yield, highlighting its importance for efficient proinsulin maturation. Essentially, PDI acts early to prevent aggregation and later to correct disulfide bonds, ensuring high-yield proinsulin folding (Winter et al., 2002). Further studies using a mutant PDI lacking the peptide binding domain (PDI-aba’c) shed light on its specific roles. This mutant PDI retained its redox potential but lacked chaperone activity due to the absence of the b' domain and its peptide binding ability (Winter et al., 2011). Interestingly, PDI-aba’c still increased proinsulin folding and yield, suggesting that in a fully oxidized environment, PDI’s primary function might be rearranging disulfide bonds, not necessarily binding to unfolded proteins. Further research is needed to fully understand the nuances of PDI function in *β*-cells and its potential as a therapeutic target for pancreatic *β*-cell dysfunction.

#### 4.1.1 PDIA1

PDIA1, a member of the PDI family facilitates the formation of correct disulfide bonds within proteins. A recent study showed that PDIA1 is essential for proper insulin production under metabolic stress conditions (Jang et al., 2019). β-cells lacking PDIA1 exhibit a range of impairments, including the accumulation of misfolded proinsulin aggregates, disruptions in the transport of vesicles from the ER to the Golgi apparatus, and a decrease in insulin granule content. These findings underscore the critical role of PDIA1 in the oxidative maturation of proinsulin within the ER. In response to PDIA1 deficiency, β-cells attempt to compensate by overexpressing BiP, possibly to refold the largely misfolded proinsulin. Overall, PDIA1’s disulfide isomerase activity helps lessen misfolded proteins and maintain ER homeostasis.

#### 4.1.2 PDIA6

Another PDI family member, PDIA6, exhibits a unique function. In a study comparing different PDIs, only PDIA6 was found to preferentially interact with misfolded *Akita* proinsulin (Gorasia et al., 2016). This interaction was confirmed in βTC-6 β-cell lines. Like other PDIs, PDIA6 possesses oxidoreductase activity and is induced under unfolded protein response (UPR) conditions. Notably, PDIA6 activity was significantly higher in cells containing misfolded *Akita* proinsulin compared to wild-type proinsulin, with expression levels increasing 10-fold (Gorasia et al., 2016). These findings suggest that PDIA6 plays a role in processing specifically misfolded proinsulin. Recent research (Yassin et al., 2024) has illuminated PDIA6’s broader role in the ER stress response, particularly within the context of selective ER retention (sERr) (Yassin et al., 2024). This process can involve the formation of large disulfide-bonded complexes, a phenomenon also observed with misfolded proinsulin. It was demonstrated that while ERp44 (another PDI family member) promotes sERr and increases the size of these complexes, PDIA6 acts antagonistically. Despite constitutively interacting with ERp44 via disulfide bonds, PDIA6 deletion slows down recovery from sERr, suggesting that PDIA6 facilitates the release of retained proteins (Yassin et al., 2024). PDIA6 may play a similar role in proinsulin proteostasis, promoting the processing or degradation of misfolded proinsulin and ensuring proper handling of the ER retained species.

#### 4.1.3 Erp72

Erp72, is involved in the PERK pathway, a signaling cascade activated during ER stress (Wang et al., 2014; Gupta et al., 2010; Feng et al., 2009). Suppression of PERK activity (by C-terminus deletion) caused a 3.3-fold increase in Erp72 protein levels (Gupta et al., 2010), which could result in impaired ERAD given Erp72 is a negative regulator of retrotranslocation (Forster et al., 2006). If the increased expression of ERp72 also increases ER retention of secretory proteins such as proinsulin, this would indicate a trafficking defect. Interestingly, ablation of PERK in INS1 832/13 β-cell lines showed a significant alteration in the redox state of Erp72 (Feng et al., 2009). This finding suggests that the protein quantity and its proper folded state with the correct redox balance are essential for Erp72 function. Additionally, manipulating Erp72 expression in PERKi cells demonstrated a partial rescue of the impaired ER function. Both overexpression and reduced activity of Erp72 showed positive effects, suggesting a complex regulatory role. However, combined siRNA knockdown of both BiP and Erp72 revealed a more dominant role for BiP in maintaining ER function (Sowers et al., 2018).

#### 4.1.4 Erp57

Similar to Erp72, Erp57 exhibits a disrupted redox state in PERKi models without changes in protein levels (Sowers et al., 2018). However, other studies suggest a different role for Erp57. Overexpression of Erp57 contributes to β-cell survival, potentially linking it to the observed autophagy in β-cells of type 2 diabetes islets. Additionally, suppression of the Erp57 led to increased levels of cleaved Caspase-3, a protein involved in apoptosis (Yamamoto et al., 2014). These findings suggest Erp57’s function in promoting β-cell survival.

### 4.2 ER oxidoreductin 1 (Ero1)

Other oxidoreductase families exist outside of the PDI family, including Ero1. Pancreatic *β*-cells express two forms of the oxidoreductase Ero1: Ero1α and Ero1β. Unlike the ubiquitously expressed Ero1α, Ero1β expression is very limited among cell types but is well expressed in pancreatic *β*-cells (Cabibbo et al., 2000; Ramming and Appenzeller-Herzog, 2012; Zito et al., 2010), suggesting that it might play a role in proinsulin folding. Both Ero1α and Ero1β contribute to proinsulin folding and can positively influence native proinsulin anterograde export through different mechanisms.

#### 4.2.1 ER oxidoreductin 1β (ERO1β)

Ero1β is a pancreas-specific disulfide oxidase (Zito et al., 2010)that is upregulated in response to ER stress. It has been observed to promote insulin synthesis in *β*-cells and contribute to glucose homeostasis (Zito et al., 2010). Overexpression of ERO1β in *β*-cells leads to improved proinsulin folding, enhanced ER-to-Golgi trafficking, and increased insulin production. However, knockout of this gene in mice did not result in severe proinsulin misfolding (Zito et al., 2010), suggesting that other oxidoreductases can compensate for its absence and provide an oxidative environment for the ER. Proinsulin misfolding and ER stress are implicated in the development and progression of diabetes. Interestingly, while ERO1β overexpression leads to increased expression of UPR genes and ER enlargement, indicating ER stress, its expression decreases in the *Akita* mouse model, which exhibits increased ER stress. This decrease might be due to the reduced β-cell mass in this model. The expression of ERO1β gradually declines with age in *Akita* mice, correlating with increased glucose intolerance (Zito et al., 2010). This contrasts with the increased expression of other UPR genes, suggesting a unique function for ERO1β in ER stress response. It is hypothesized that decreased ERO1β expression might contribute to the progression of diabetes and β-cell dysfunction by exacerbating ER stress. However, manipulating ERO1β expression, such as overexpression, might not ameliorate β-cell function and could even worsen ER stress (Awazawa et al., 2014). These findings highlight the importance of maintaining a balanced level of ERO1β to preserve ER homeostasis and β-cell function for insulin synthesis.

#### 4.2.2 *ER* oxidoreductin *1α (Ero1α)*

In contrast to ERO1β, oxidoreductase Ero1α is ubiquitously expressed in various cell types. Increasing the expression of Ero1α can limit ER stress by enhancing ER oxidation and modifying the folding of proinsulin. Studies carried out by (Wright et al., 2013) (Wright et al., 2013) has shown that different variants of Ero1α, including wild-type and variants lacking regulatory disulfides, can modify the folding of misfolded proinsulin-G(B23)V mutant, leading to its secretion. Overexpression of Ero1α can limit proinsulin misfolding and its negative consequences. This increase is beneficial for exporting wild-type proinsulin in cells that express both wild-type and misfolded mutant proinsulin (Wright et al., 2013), emphasizing Ero1α′s ability of tone down the dominant negative effect of the mutant over the wild-type proinsulin. These properties make Ero1α a potential therapeutic target for MIDY.

### 4.3 Peroxiredoxin 4 (PRDX4)

PRDX4, an ER-localized protein has been reported to interact with both native and misfolded proinsulin, facilitating proper disulfide bond formation (Tran et al., 2020). A study by (Tran et al., 2020) (Tran et al., 2020) revealed that PRDX4’s interaction with the ER chaperone BiP is independent of proinsulin folding, and PRDX4 binding to proinsulin is independent of BiP, which suggests complex interactions between these proteins in the ER. The work also explores the impact of glucose levels on PRDX4 activity (Tran et al., 2020). While high glucose levels increase insulin secretion in Min6 cells, they also lead to increased sulfonylation of PRDX4. Sulfonylation-mediated inactivation of PRDX4 could reduce its ability to protect proinsulin from oxidative damage and misfolding. However, in human islets, the increase in glucose does not significantly affect PRDX4 sulfonylation (Tran et al., 2020). This difference raises questions about the role of PRDX4 in T2D patients and its potential as a biomarker for oxidative stress in *β*-cells. Overall, PRDX4 appears to influence proinsulin proteostasis; however, more research is needed to elucidate its potential implications in diabetes.

## 5 Proinsulin anterograde trafficking

### 5.1 ER-to-Golgi trafficking

Beyond genetic mutations in the INS gene and dysfunctions of chaperones and oxidoreductases, impaired ER-to-Golgi trafficking can significantly impact proinsulin folding and aggregation. A complex machinery, including proteins like Sar1, Sec23, Sec24, Sec13, and Sec31, is involved in the formation of COPII vesicles, which mediate this transport (Fromme et al., 2008; Tang and Ginsburg, 2023). The small GTPase Sar1 plays a pivotal role in regulating coat assembly and disassembly. This trafficking step, while occurring after proinsulin folding, can exert a negative feedback loop on ER homeostasis, potentially increasing misfolded proinsulin (Arunagiri et al., 2024). Studies using non-reducing SDS-PAGE/immunoblotting have revealed the formation of proinsulin oligomers associated with impaired ER-to-Golgi transport (Arunagiri et al., 2024). Brefeldin A (BFA) is a well-known pharmacological inhibitor of ER-to-Golgi transport. It indirectly restricts the association of the COPI coat. When cultured β-cells or purified human/rodent islets were treated with BFA, it led to the accumulation of misfolded proinsulin in the ER (Arunagiri et al., 2024; Zhu et al., 2019). Likewise, the mutations in genes encoding proteins involved in COPII vesicle formation, such as Sar1 or YIPF5, can disrupt ER-to-Golgi trafficking. While the YIPF5-I98S mutation, implicated in neonatal diabetes, impairs proinsulin trafficking from the ER to the Golgi leading to proinsulin misfolding in stem cell-derived *β*-cell-like clusters (Arunagiri et al., 2024; De Franco et al., 2020), the Sar1 mutation or deficiency in *β*-cells has been shown to cause proinsulin misfolding, trafficking and maturation defects (Zhu et al., 2019; Fang et al., 2015). The defective ER export is hence proposed to be a major contributor to ER stress induced by misfolded proinsulin. A recent work supporting these findings came from a study centered on IER3IP1, a protein involved in proinsulin ER-to-Golgi trafficking. IER3IP1 is highly expressed in β cells, and mutations in IER3IP1 can lead to impaired proinsulin trafficking, ER stress, and β-cell dysfunction (Yang et al., 2022). Moreover, IER3IP1 mutations (such as the IER3IP1V21G variant) can cause proinsulin accumulation in the ER, reduced insulin secretion, and increased ER stress. In agreement with this, the CRISPR-Cas9-mediated IER3IP1 KO cells exhibited impaired proinsulin trafficking, as evidenced by reduced colocalization of proinsulin with Golgi markers and increased ER retention. The knockout model also showed an increased ER stress. Overall, these findings highlight the importance of proper ER-to-Golgi trafficking for proinsulin proteostasis and insulin production in β-cells.

### 5.2 Golgi export of proinsulin

Recent work by Boyer et al. (2023) has highlighted the importance of synchronized proinsulin trafficking and the impact of delayed Golgi export on β-cell function and diabetes pathogenesis. The concept of synchronized trafficking emphasizes the coordinated movement of proinsulin through the secretory pathway, from ER exit to Golgi transit, insulin granule formation, and ultimately secretion. This study, using the novel proCpepRUSH reporter and RUSH system, provides a beautiful example of visualizing and quantifying this synchronization, revealing the precise sequence of key events. Specifically, they observed proinsulin’s progression from the ER to the Golgi, followed by its condensation within the Golgi, and subsequent appearance in mature insulin granules, mirroring the known timeline of proinsulin processing (Boyer et al., 2023). Interestingly, this research demonstrates defects in proinsulin export from the Golgi in both dietary (Western diet) and genetic (db/db) models of diabetes. In these models, proinsulin accumulates in the Golgi, evidenced by increased proCpepRUSH signal proximal to the Golgi, and TIRF microscopy confirmed a decrease in proinsulin at the plasma membrane, suggesting impaired insulin granule delivery. This delay is linked to significant structural changes in the Golgi, including dilated cisternae, fewer cisternae per stack, and increased vesiculation, observed using electron microscopy (Boyer et al., 2023). These structural alterations likely disrupt proinsulin processing and packaging, as the study showed proCpepRUSH processing occurs post-Golgi exit, within the maturing granules. The extended chase experiments in the study further revealed that while the initial Golgi export was delayed, the proinsulin eventually reached its final destination, suggesting a kinetic delay rather than a complete block (Boyer et al., 2023). Thus, by highlighting both the synchronized nature of proinsulin trafficking and the specific vulnerability of Golgi export, this work emphasizes the complex relationship between these processes and their importance for glucose homeostasis, suggesting the Golgi as a potential target for diabetes interventions.

## 6 Proinsulin intracellular degradation

Misfolded proinsulin accumulation can lead to ER stress and impaired *β*-cell function. Hence, several degradation pathways work in concert to eliminate misfolded proinsulin.

### 6.1 *ER-*Associated *degradation (ERAD)*

ERAD is a highly regulated process that targets misfolded proteins for degradation by the proteasome (McCracken and Brodsky, 2003; Krshnan et al., 2022). Misfolded proinsulin in the ER should befirst unfolded, and chaperoned to the ERAD complex (by BiP) to be then transported back to the cytosol (retrotranslocation) and marked for proteasomal degradation via ubiquitination. These steps essentially describe the ERAD of proinsulin in *β*-cells. Derlin-2, HRD1, and p97 are key components of the ERAD pathway (Hoelen et al., 2015). The knockdown of these proteins leads to increased proinsulin levels, suggesting their involvement in its degradation. Furthermore, SEL1L, another ERAD component, has been shown to play a critical role in proinsulin processing and degradation (Hu et al., 2019). SEL1L-deficient pancreatic β-cells exhibit impaired proinsulin processing and increased degradation of wild-type and folding-deficient mutant proinsulin. These findings highlight the importance of ERAD in maintaining proinsulin homeostasis. Moreover, acute inhibition of the proteasome (using MG132) leads to increased proinsulin levels and accumulation of misfolded proteins in the ER, further supporting the role of proteasomal degradation in proinsulin quality control (Xu et al., 2022). Recent research has also unveiled a link between proinsulin degradation and the development of autoimmunity. The ERAD enzyme UBE2G2 is involved in proinsulin degradation and the subsequent presentation of the PPIB10-18 autoantigen (Cremer et al., 2024). Misfolded proinsulin triggers UPR, leading to ERAD-mediated dislocation of proinsulin (to relieve ER stress). By degrading proinsulin, ERAD machinery (HRD1/UBE2G2 ubiquitination complex) may contribute to the generation of proinsulin B-chain autoantigens, which can trigger autoreactive CD8^+^ T cell responses (Cremer et al., 2024). This suggests that reducing the proinsulin degradation load on ERAD, potentially by improving the β-cell’s oxidative folding environment, could help alleviate the risk of autoimmunity. Collectively, these studies provide valuable insights into the complex mechanisms regulating proinsulin degradation in pancreatic *β*-cells.

### 6.2 Autophagy

Autophagy, a cellular process that involves the formation of autophagosomes to engulf and degrade cellular components, including misfolded proteins, can be activated as a compensatory mechanism when the ERAD pathway is compromised. Chloroquine treatment of purified rodent islets resulted in the accumulation of misfolded proinsulin, suggesting that autophagic pathways may be involved in the disposal of misfolded proinsulin (Shrestha et al., 2023). More recent studies surprisingly showed that chloroquine exposure on pancreatic β-cells led to proinsulin misfolding and its accumulation in the ER (Xu et al., 2024) instead of autophagosomes. This raises questions about the precise role of autophagy in proinsulin clearance and the specificity of chloroquine’s effects in β-cells (discussed in Section 9.3c).

### 6.3 ER-Phagy

ER-phagy, also known as reticulophagy, is a specialized form of autophagy for eliminating misfolded proinsulin. When misfolded proinsulin aggregates form, chaperones like Grp170 attempt to bind and prevent further aggregation (Cunningham et al., 2017). If this fails, ER-phagy kicks in. Here, a protein called Reticulon-3 (RTN3) plays a critical role (Cunningham et al., 2019). However, studies suggest RTN3 might not have direct access to the part of the ER (ER lumen) where proinsulin resides. This is where another protein, PGRMC1, comes in. Located within the ER lumen, PGRMC1 acts as a bridge between misfolded proinsulin and RTN3, facilitating their interaction and subsequent degradation by lysosomes. Notably, PGRMC1 exhibits size selectivity, preferentially targeting smaller misfolded mutant proinsulins (Chen et al., 2021). Chemically interfering with PGRMC1 function was found to partially restore insulin storage capacity by blocking the ER-phagic degradation pathway for both wild-type and mutant forms of proinsulin (Chen et al., 2021).

A well-coordinated functioning of ERAD, autophagy, and ER-phagy ensures the elimination of misfolded or excess proinsulin in *β*-cells. Understanding these degradation pathways can provide insights into the pathogenesis of diabetes and identify potential interventions.

## 7 Role of mitochondria in proinsulin proteostasis

**FIGURE 6:**
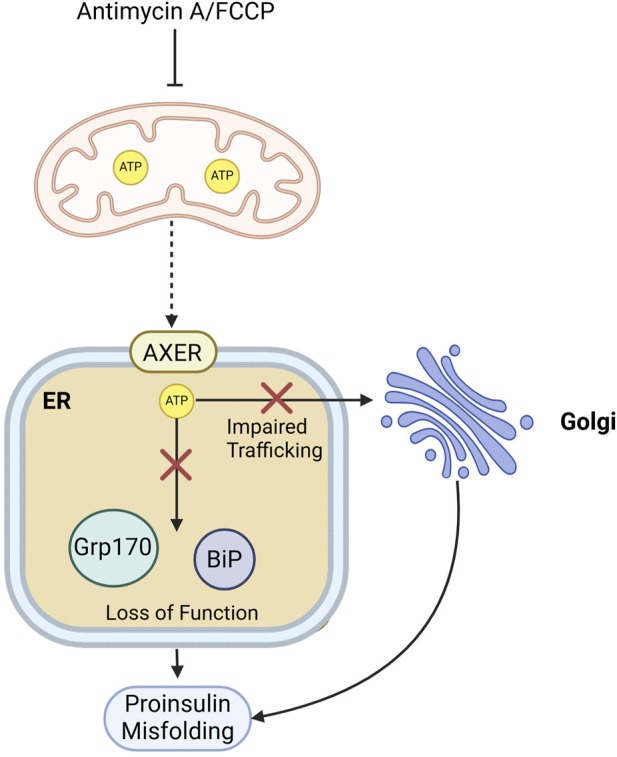
Mitochondrial energy supply supports proinsulin folding and ER-to-Golgi transport. Mitochondrial ATP depletion in β-cells by Antimycin A or FCCP treatment, could impact proinsulin folding by affecting the function of ATP-dependent ER chaperones like BiP or by impairing proinsulin trafficking from the ER to the Golgi.*Red crosses indicate blocked pathways, and dashed arrow indicated impaired mitochondria-to-ER ATP shuttling.*

Mitochondria are important for maintaining proinsulin proteostasis in *β*-cells. By regulating cellular redox donor cycles, particularly NADPH and thioredoxin, mitochondria influence the redox environment of the ER. Recent research by (Rohli et al., 2024) revealed that proper mitochondrial function is crucial for maintaining ER redox balance, which supports NADPH-dependent redox cycles essential for proinsulin folding and trafficking (Rohli et al., 2024). The authors find that mitochondrial dysfunction and metabolic inhibition lead to ER hyperoxidation. NADPH and thioredoxin are vital in sustaining ER redox homeostasis, and suppressing thioredoxin-interacting protein (Txnip) can restore ER function and proinsulin trafficking. Moreover, it is proposed that β-cell deterioration in T2D is associated with both mitochondrial dysfunction and redox imbalance, affecting proinsulin maturation and insulin granule stores, contributing to diabetes progression (Rohli et al., 2024). Targeting redox pathways, particularly through antioxidant interventions like TXNIP inhibition, is suggested as a means to enhance β-cell health and function.An independent study by Arunagiri, et al. (2024) pointed out that the blocking of ATP production in the mitochondria using Antimycin A or FCCP in Min6 cells leads to proinsulin misfolding (Arunagiri et al., 2024), most likely due to the unavailability of ATP for the ER chaperone functioning. ATP is known to shuttle from mitochondria to the ER via an ER membrane channel called AXER (Klein et al., 2018; Yong et al., 2019). In addition to ATP supply to the ER, ATP production is also important for the anterograde trafficking of the proteins in cells (Jamieson and Palade, 1968). Depletion of ATP in *β*-cellscould therefore potentially impair proinsulin ER-to-Golgi trafficking, and this trafficking defect in turn might cause a folding problem (Figure 6), as described in section 5. Targeting the ER-mitochondrial interactions may offer a potential approach for managing type 2 diabetes by improving β-cell function.

## 8 Extrinsic factors

Beyond the intrinsic cellular factors discussed in the preceding sections, extrinsic factors, including dietary intake and prescribed medications, can significantly impact proinsulin proteostasis and subsequent insulin biosynthesis within *β*-cells.

### 8.1 Nutrient/diet

Nutrient availability, particularly during feeding cycles significantly impacts proinsulin synthesis. Rapid dephosphorylation of the translation initiation factor eIF2α, induced by nutrient intake, precedes a surge in proinsulin levels. Conversely, rephosphorylation of eIF2α during fasting periods correlates with a decrease in proinsulin levels (Xu et al., 2023). Subthreshold genetic predisposition to proinsulin misfolding can remain silent until triggered by a high-fat diet (HFD). Male mice with this predisposition (carrying a MIDY-like INS gene mutation, R (B22)E) exhibit glucose intolerance and rapidly develop frank diabetes upon HFD exposure, but do not develop diabetes when on a regular diet (Alam et al., 2021). A rapid increase in proinsulin misfolding and a decline in insulin production in the model are associated with dramatic pancreatic pathology (Alam et al., 2021). These findings underscore the critical role of dietary factors in regulating proinsulin synthesis folding, and, ultimately, insulin production. Identifying the mechanisms protecting females from diabetes in this model could offer novel preventative strategies for both sexes.

### 8.2 Hormones and drugs

#### 8.2.1 Estrogen

The protective effect of estrogen against diabetes in women has been previously reported, but the underlying mechanisms remained unclear. To address this, (Xu et al., 2018) administered Conjugated Estrogens (CE) in *Akita* mice (Xu et al., 2018). CE, through its primary receptor ERα, was suggested to stabilize the ERAD pathway and reduce ER stress in β-cells. ERAD degraders, such as HRD1 and SEL1L, prevent the premature degradation of functional proteins. CE activation of ERα stabilizes these proteins by inhibiting their proteasomal degradation mediated by UBC6e, the only ubiquitin-conjugating enzyme localized to the ER. When ERα is absent, UBC6e expression increases, and this causes the degradation of ERAD components and the accumulation of misfolded proinsulin. To sum up, estrogen restores *β*-cell function by modulating the proinsulin degradation pathway (Xu et al., 2018).

### 8.3 Olanzapine

Olanzapine, a medication commonly used to treat schizophrenia, can have unexpected consequences beyond its intended therapeutic effects. In addition to its well-known association with weight gain, olanzapine can impede the normal function of pancreatic *β*-cells. By interfering with the normal folding and processing of proinsulin, olanzapine was reported to impair insulin production by unknown mechanisms (Ninagawa et al., 2020). This observation suggests that olanzapine administration can lead to the development of diabetes, even in patients who do not experience significant weight gain, a more commonly recognized side effect of the drug.

### 8.4 Chloroquine

Chloroquine, a common antimalarial medication, also affects cellular processes beyond its intended use. In a study by (Xu et al., 2024), it was found that chloroquine disrupts the proper folding of proinsulin in the pancreatic β-cells, causing misfolded proinsulin to accumulate in the ER. This accumulation was not due to autophagy inhibition or chloroquine’s pH effects but resulted from impaired interaction between proinsulin and PDI (Xu et al., 2024). Consequently, the production of mature insulin is significantly decreased, potentially impairing glucose metabolism and, in severe cases, contributing to diabetes development. These findings highlight the need for caution with chloroquine in diabetes treatment. Its effects on pancreatic β-cells necessitate further investigation to understand its safety and efficacy.

## 9 Proinsulin folding landscape in the context of native function and dysfunction

Considering the importance of proinsulin proteostasis in β-cells, exploring the interplay between protein structure and disease becomes crucial. This naturally leads to the question: What is the structural basis of proinsulin misfolding in diabetes? This is an area of active investigation, with biophysical techniques like NMR, X-ray crystallography, and molecular dynamics (MD) simulations playing a vital role in uncovering the underlying protein dynamics.

### 9.1 Inter- and intra-chain disulfide bonds and their roles in proinsulin folding and ER export

The B- and A-chains of proinsulin form the insulin core, which is stabilized by three disulfide bonds: (i) A6–A11 (intra-A-chain), (ii) A7–B7 (inter-chain), and (iii) A20–B19 (inter-chain, stabilizing the insulin core). These disulfide bonds must form in a sequential and cooperative manner to ensure proper folding. Incorrect disulfide pairing can result in folding defects and ER retention as indicated earlier. The folding process of proinsulin is guided by a combination of intramolecular disulfide bond formation, C-peptide-mediated structural organization (Ksenofontova et al., 2014; Jung et al., 2017), and interactions with other proteins and chaperones. Insights from NMR unveil that most crystal structure hydrogen bonds in insulin are transient in solution, with stable bonds primarily around the B19-A20 disulfide bridge, a potential folding nucleus (Hua et al., 2011; Gorai and Vashisth, 2022). The Cys (B19)–Cys (A20) bond plays a pivotal role in facilitating the formation of other disulfide bridges (Haataja et al., 2016). Given the importance of these disulfide bonds in proinsulin folding, it is crucial to examine their specific roles in secretion. Notably, the Cys (B7)–Cys (A7) and Cys (B19)–Cys (A20) bonds are essential for export from the ER. While the Cys (A6)–Cys (A11) bond is crucial for insulin stability, it is dispensable for proinsulin export (Haataja et al., 2016), and mutations lacking this bond still achieve efficient secretion.

### 9.2 Proinsulin B- and A-chain substitutions influence proinsulin folding

MD simulations have been instrumental in revealing the remarkable flexibility of insulin’s B-chain (Papaioannou et al., 2015). They pinpoint how the B-chain switches between its active (T-state) and inactive (Rf-state) conformations (Kosinova et al., 2014), highlighting the significance of localized interactions and a hinge mechanism involving Phe24 in orchestrating receptor binding (Papaioannou et al., 2015). The N-terminal segment of the B-chain (B1–B8) plays a critical role in guiding disulfide bond formation. Studies show that mutations in this region, such as PheB1 deletion, impair folding efficiency and increase misfolding rates (Liu et al., 2010c). The inability to adopt native conformations due to mutations or mispaired disulfide bonds could result in loss of folding efficiency resulting in misfolding. Mutations affecting PheB24 (e.g., PheB24 → TyrB24) impair folding, despite having minimal effects on insulin receptor binding (Rege et al., 2020).

In MIDY, the aberrant B-chain interactions are responsible for the dominant-negative effect of MIDY mutant on WT proinsulin (Sun et al., 2020) (discussed in section 2). The B9-B19 α-helix (residues 8–29), normally facilitating proinsulin homodimerization for proper processing, becomes a site of pathological heterodimerization in MIDY (Sun et al., 2020). Mutations like C(A7)Y lead to misfolding, creating a mutant proinsulin that interacts with wild-type proinsulin, disrupting its ER export and processing. Importantly, Tyr-B16, a key residue in the dimerization interface, is essential for this pathological interaction. While mutations like Y(B16)D/A impair homodimerization, they also weaken the mutant-wild-type interaction when combined with a MIDY mutation (Sun et al., 2020). Disruption of the α-helix (Y(B16)P) impairs folding but does not cause the same dominant-negative effect as the *Akita* mutation, highlighting the specific nature of the pathogenic interaction. The misfolded MIDY mutant, via its B-chain including Tyr-B16, “hijacks” wild-type proinsulin, preventing its maturation and causing insulin deficiency (Sun et al., 2020).

The A-chain of proinsulin consists of two α-helices (A1–A8 and A12–A20), which remain largely conserved during the transition from proinsulin to insulin (Yang et al., 2010). However, the A-chain exhibits significant local flexibility in proinsulin, particularly around the A1–A8 segment, which does not assume a fully stable α-helical conformation in the precursor molecule. This flexibility likely facilitates proper folding and disulfide bond formation, ensuring that proinsulin reaches its native state efficiently before cleavage into insulin. The A-chain participates in all three disulfide bonds in proinsulin (B19-A20, B7-A7 and A6-A11). These bonds help stabilize the structure, preventing misfolding and aggregation. However, mutations affecting cysteine residues in the A-chain such as the *Akita* mutation C(A7)Y can lead to incorrect disulfide pairing, resulting in proinsulin misfolding and retention in the ER (Zuber et al., 2004) and β-cell dysfunction, and the C (A6)S MIDY mutation results in loss of the A6-A11 intra-chain bond, destabilizing the α-helices of the A-chain. Likewise, a mutation that might disrupt the A20-B19 inter-chain bond would not only impair insulin core stability and receptor-binding ability, but also significantly affect proinsulin folding.

Mutations at key hydrophobic residues within the A-chain (such as A2, A8, A13, and A19) affect the packing of α-helices, causing (a) Reduced structural integrity of the insulin core, and (b) Lower affinity for the insulin receptor due to altered surface properties. For instance, V(A3)L mutation would destabilize A-chain helix, impairing receptor interactions and reducing insulin bioactivity (Nishi and Nanjo, 2011). While mutations at the interface between the A- and B-chains, which can disrupt intermolecular hydrogen bonding and van der Waals interactions, specific mutation on the A-chain specifically could have different outcomes on proinsulin structure and trafficking. For example, Y(A19)C increases misfolding risk and causes partial ER retention of the protein (Rajan et al., 2010). On the other hand, mutating E(A17) disrupts protein sorting to the regulatory secretory pathway, indicating this A-chain residue is critical for efficient sorting (Dhanvantari et al., 2003).

### 9.3 The structural implication of the flexible C-peptide

Proinsulin and insulin share a key ability to form hexamers in the presence of zinc, which is crucial for efficient storage and secretion (Pekar and Frank, 1972). However, proinsulin hexamers are more dynamic than insulin hexamers, likely due to the structural interference of the C-peptide. This dynamic equilibrium affects the rate of proinsulin processing and insulin secretion. Unlike insulin, which is prone to amyloid fibrillation, proinsulin is highly resistant to fibrillation (Huang et al., 2005), and this protection is provided by the C-peptide, which prevents the β-sheet stacking required for amyloid formation (Huang et al., 2005). Crystallographic studies confirm that the insulin core in proinsulin adopts a conformation similar to mature insulin (Frank et al., 1972), though with greater flexibility due to the presence of the C-peptide, which aids in the alignment of folding intermediates (Jung et al., 2017). This mobility of the C-peptide facilitates the crucial pairing of cysteine residues for disulfide bond formation, essential for the correct folding of insulin’s A- and B-chains (Ksenofontova et al., 2014; Jung et al., 2017). The C-peptide guides correct folding but do not participate in insulin receptor binding, making it dispensable after proinsulin is processed into insulin. Evolutionary analysis of the proinsulin sequences from mammals, reptiles and birds by Landreh and Jornvall (2015) reveal a remarkable evolutionary and structural flexibility of the C-peptide, which exhibits a much higher sequence variation than other regions of the protein (Landreh and Jornvall, 2015). The A- and B-chains of insulin on the other hand exhibit a high degree of evolutionary conservation across species (Landreh and Jornvall, 2015). This difference in conservation levels influences the structural role, biochemical interactions, and evolutionary flexibility of C-peptide, allowing proinsulin to behave differently from insulin. The C-peptide is also home to fewer disease mutations compared to the A- and B- chains which show tight evolutionary conservation.

## 10 Discussion and future perspectives

**FIGURE 7:**
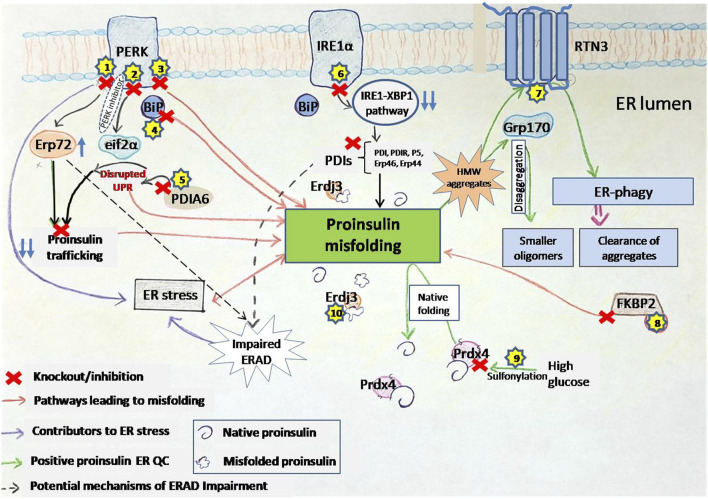
Orchestrating proinsulin proteostasis in β-cells: Molecular Chaperones, oxidoreductases, and the UPR. (1) PERK ablation, leading to Erp72 upregulation, may impair proinsulin degradation or trafficking. Independently, PERK inhibition can induce ER stress in β-cells, (2) PERK inhibition (by PERKi) prevents eIF2α phosphorylation, negatively impacting the UPR primarily through dysregulation of protein synthesis, (3) Treatment of β-cells with PERKi for 10–12 h induces proinsulin misfolding, (4) BiP inactivation (by SubAB or PERK inhibition) impairs proinsulin folding in β-cells, (5) PDIA6 deletion may impair both the UPR and proinsulin trafficking; Defective proinsulin trafficking could hinder proinsulin folding in the ER, (6) Inhibition of the IRE1α-XBP1 pathway deregulates PDI activity in β-cells, potentially impeding proinsulin folding or disrupting ERAD through impaired PDI-mediated retrotranslocation, (7) Grp170 either directly targets mutant high-molecular weight (HMW) proinsulin aggregates or collaborates with reticulon 3 (RTN3), which engages with PGRMC1 on the ER luminal side (not shown), to clear aggregates via ER-phagy in the cytosol, (8) FBKBP2 knockout results in proinsulin misfolding, (9) Highglucose-induced sulfonylation of PRDX4 impairs its protective function against proinsulin misfolding, (10) Erdj3 may promote proinsulin folding by acting as a co-chaperone with BiP.

This review enunciates the complex network of genetic and cellular factors governing proinsulin proteostasis in pancreatic β-cells (Figure 7; Table 1) and its crucial role in diabetes. INS gene mutations, especially those impacting cysteine residues and conserved regions, directly impair proinsulin folding and disulfide bond formation, often exhibiting a dominant-negative effect (Liu et al., 2010a; Haataja et al., 2021). This underscores the importance of ER quality control mechanisms, involving a network of chaperones (BiP, ERdjs, Grp170, FKBP2) and oxidoreductases (PDIs and Ero1s) that facilitate proper folding, prevent aggregation, and target misfolded proinsulin for degradation. Disruptions to these systems, whether through genetic mutations, ER stress, or impaired ER-to-Golgi trafficking, lead to proinsulin accumulation and *β*-cell dysfunction. The UPR, particularly the PERK and IRE1α-XBP1 pathways, emerges as a central regulator, balancing protein synthesis with ER stress management. Notably, PERK inhibition’s delayed effect on proinsulin aggregation suggests downstream effects on chaperone function, especially BiP (Sowers et al., 2018). On the other hand, the IRE1α-XBP1 pathway’s regulation of PDI expression highlights the importance of oxidative folding regulated through UPR elements (Tsuchiya et al., 2018). Furthermore, the collective action of ERAD, autophagy, and ER-phagy demonstrates a sophisticated yet vulnerable protein quality control system in β-cells. Mitochondrial health and ER redox state add further complexity, suggesting that targeting mitochondrial dysfunction could indirectly improve proinsulin folding. Extrinsic factors like diet, estrogen, and medications like olanzapine and chloroquine further emphasize the vulnerability of proinsulin proteostasis (Xu et al., 2023; Alam et al., 2021; Xu et al., 2018; Ninagawa et al., 2020). The discovery of OSGEP as a key regulator of proinsulin translation and ER stress homeostasis (Liu et al., 2024) along with the paradoxical overexpression of FKBP2 in T2D (Hoefner et al., 2023), present compelling avenues for future research in diabetes intervention. Likewise, the potential link between ERAD and autoimmunity (Cremer et al., 2024) suggests a connection between proinsulin misfolding and T1D, warranting further exploration.

**TABLE 1:** A summary of representative factors influencing proinsulin proteostasis in β-cells.

Factors	Role in proinsulin proteostasis	Model organisms or cell lines used	Key references and further reading
*INS gene mutations*	Mutations in the INS gene can disrupt proinsulin folding, leading to misfolded mutant proteins that can form harmful aggregates and trap healthy proinsulin molecules within β-cell ER, ultimately impairing insulin production and contributing MIDY	Rat or mouse pancreatic β-cell lines (INS1 cells, Min6 cells), HEK 293T cells, Akita mouse model, mouse islets	Liu et al. (2010a), Sun et al. (2020), Haataja et al. (2021), Liu et al. (2010b)
*ER Chaperones*			
BiP	• BiP is upregulated in response to ER stress likely caused by misfolded proinsulin • BiP interacts with misfolded proinsulin • Disruption of BiP function leads to proinsulin aggregation	C57BL/KsJ db/db mice, mouse pancreatic islets, human pancreatic islets, rat pancreatic β-cell lines	Laybutt et al. (2007), Arunagiri et al. (2019), Liu et al. (2005), Wang et al. (1999)
ERdj3	ERdj3 interacts with both wild-type and mutant proinsulin, potentially by assisting BiP in proinsulin quality control	Insulinoma cell lines	Gorasia et al. (2016)
Grp170	• Grp170 specifically targets aggregated mutant proinsulin for degradation • Working in concert with RTN3, Grp170 manages proinsulin aggregates	Rat INS-1 832/13 cell line, HEK 293T cells	Cunningham et al. (2017), Cunningham et al. (2019)
FKBP2	• FKBP2 is crucial for early proinsulin folding and prevents aggregation • Isomerization at proinsulin P28 by FKBP2 facilitates release from misfolded states	Rat insulinoma INS-1E cells	Hoefner et al. (2023)
*Unfolded Protein Response*			
PERK	• PERK-mediated eIF2α phosphorylation regulates proinsulin translation • PERK influences BiP function, impacting proinsulin aggregation • PERKi treatment of β-cell or islets leads to proinsulin misfolding	HEK 293T cells, INS1E cells	Arunagiri et al. (2019), Sowers et al. (2018), Harding et al. (2012)
IRE1α–XBP1 Pathway	IRE1α–XBP1 pathway is essential for maintaining β-cell function and insulin production. This pathway regulates the expression of PDIs crucial for proinsulin folding	INS1 832/13 and Min6 cells, mouse models, mouse pancreatic islets, human pancreatic islets, Rat pancreatic β-cell lines INS1E and INS-832/13	Tsuchiya et al. (2018)
Proinsulin Synthesis	Glucose stimulates proinsulin synthesis. Increased proinsulin synthesis can overwhelm the ER’s folding capacity, leading to misfolding and UPR induction	Rat INS1 832/13 and mouse Min6 cells, rat Islets	Sowers et al. (2018), Harding et al. (2012), Ward et al., 1987; Leahy (1993), Uchizono et al. (2007), Halban et al. (1983)
ER Calcium Homeostasis	• Deficiency of ER calcium pump, SERCA2, impairs calcium signaling, proinsulin processing, and insulin secretion • PERK inhibition interferes with calcium fluxes and proinsulin folding in β-cells	βSERCA2KO mice, INS-1 cell line, Human pancreatic islets, INS1 832/13 and Min6 cells, Islets from C57BL6/J mice	Sowers et al. (2018), Iida et al. (2023), Wang et al. (2013)
*Oxidoreductases*			
PDI	PDIs are crucial for proper disulfide bond formation in proinsulin. Independent studies on PDI family members have suggested a positive influence of the protein on proinsulin trafficking. Representative PDI family members and their reported functions in β-cells are listed below • PDIA1 deficiency leads to misfolded proinsulin aggregates and decreased insulin secretion • PDIA6 interacts with misfolded proinsulin and has higher activity in cells containing this misfolded protein • Erp72 function depends on both its quantity and proper folded state with the correct redox balance. It is involved in the PERK stress signaling pathway	INS1 832/13 and Min6 cells, 293 and HepG2 cells, HEK 293T cells, C57BL/6 mice	Sowers et al. (2018), Rajpal et al. (2012), Jang et al. (2019), He et al. (2015)
Ero1β	• Ero1β promotes proinsulin folding and insulin synthesis • While reduced Ero1β expression can mitigate the effects of misfolded proinsulin in cultured Min6 cells, this protective effect is not observed in pancreatic islets *in vivo* • Ero1β expression is complexly regulated in diabetes	INS1 and 293 cells, 293 and HepG2 cells, Min6 cells, *Akita* mice, Islets from wild-type and ERO1-β mutant mice	Rajpal et al. (2012), Zito et al. (2010)
Ero1 α	Ero1α improves WT and mutant proinsulin folding and secretion	293T cells, rat insulinoma cells	Wright et al. (2013)
*Extrinsic factors*			
Nutrient/Diet	• Nutrient cycles regulate proinsulin synthesis via eIF2α phosphorylation • A high-fat diet can trigger diabetes in genetically predisposed rodent model by exacerbating proinsulin misfolding	Rat or mouse pancreatic β cell lines, HEK 293T cells, mouse islets	Xu et al. (2023), Alam et al. (2021)
Estrogen	Estrogen, acting via ERα, protects against diabetes by stabilizing the ERAD pathway, preventing premature degradation of functional proteins and mitigating ER stress caused by misfolded proinsulin	Male and female heterozygous *Akita*, Mouse islet, Human islets	Xu et al. (2018)
Olanzapine	Olanzapine interferes with proper disulfide bond formation in proinsulin, leading to misfolding and ER retention	MIN6 cells, Male BALB/c mice, mouse pancreatic islets	Ninagawa et al. (2020)
Chloroquine	Chloroquine disrupts proinsulin folding in β-cells by impairing proinsulin-PDI interaction	Pancreatic β-cells	Xu et al. (2024)

INS gene mutations result in mispaired or unpaired cysteines lead to ER retention of misfolded proinsulin, triggering ER stress (Liu et al., 2010a; Haataja et al., 2021) and β-cell dysfunction—a hallmark of MIDY (discussed in section 2). Similar misfolding involving aberrant disulfide bonding (mostly intermolecular) has been observed in WT proinsulin when the ER oxidative folding environment is unfavorable (Arunagiri et al., 2024). Modifying these disulfide interactions could therefore potentially improve native folding or alleviate dominant-negative effects (in case of MIDY mutants) and improve insulin biosynthesis and secretion, offering exciting restorative possibilities.

The importance of disulfide bonds extends beyond proinsulin. Crystallins, essential for lens transparency, rely on precise disulfide formation for stability. Similar to proinsulin, misfolded crystallins with aberrant disulfide bonds aggregate, leading to cataracts (Serebryany et al., 2024). Mutations in γD-crystallin, for example, have been linked to non-native disulfide bonding, causing structural destabilization and cataractogenesis (Serebryany et al., 2018). These parallels highlight the importance of precise cysteine pairing in protein folding and function (Narayan, 2021). The “combinatorial” nature of disulfide bonds in protein sequences with multiple cysteines underscores their importance in diverse biological systems. Incorrect pairing in crystallins, as in proinsulin, compromises functionality, emphasizing the universal challenges of achieving the correct disulfide topology.

Advances in recombinant insulin production leverage insights into proinsulin structure. Studies on artificial leader peptides fused to proinsulin have shown that subtle sequence variations can significantly impact folding, cleavage efficiency and expression levels in bacterial systems (Jung et al., 2017). Computational modeling approaches predict how these sequences affect protease accessibility and insulin maturation, offering promising strategies to enhance production (Jung et al., 2017).

The insights into the structural basis of proinsulin misfolding and the multifaceted interaction of cellular factors influencing proteostasis pave the way for novel interventions strategies. Targeting disulfide redox systems in particular, offers a promising approach for mitigating proinsulin misfolding in β-cells.

Traditional models used in β-cell biology, typically involving animal cells or immortalized cell lines (shown in Table 1), often do not accurately replicate human β-cell physiology. This shortcoming significantly impedes the translation of research into effective diabetes therapies. Human β-cells exhibit unique features in gene expression, signaling pathways, and pancreatic islet interactions that animal models and cell lines cannot fully emulate. While useful for preliminary studies, these simplified models do not capture the complex environment and responses of human β-cells *in vivo*.

To address this translational gap, researchers are increasingly turning to human-derived tissues. Stem cell-derived β-cell-like clusters (SC-βLCs), derived from pluripotent stem cells, offer a more physiologically relevant 3D model. These clusters demonstrate key β-cell functions, such as glucose-stimulated insulin secretion, and show great promise for drug screening, disease modeling, and potential cell replacement therapies. They provide a controlled and scalable system compared to primary human tissue. An example SC-βLCs is breifly highlighted in proinsulin anterograde trafficking (Section 5). Additionally, human pancreatic tissue slices and isolated islets, sourced from deceased donors with ethical adherence, offer insights into native β-cell function within their natural microenvironment. Studying these tissues enables researchers to explore β-cell interactions with other islet cell types, modulation by surrounding tissue architecture, and disruptions in conditions like type 2 diabetes. These tissues present a more complex and realistic picture of β-cell physiology than *in vitro* models.

By prioritizing human-derived models—SC-βLCs, pancreatic tissue slices, and isolated islets—β-cell biologists are making significant strides in understanding human β-cell physiology. This research is crucial for developing more effective and targeted diabetes therapies. These models facilitate a deeper understanding of disease mechanisms, identification of novel drug targets, and development of personalized medicine approaches tailored to individual patient needs. Ultimately, this shift toward human-relevant models promises to accelerate the development of better treatments and improve the lives of those with diabetes.

The complex challenge of proinsulin proteostasis demands a holistic and integrated approach for future therapies. This necessitates investigations in human tissues and the development of interventions that target not only the proinsulin protein itself, but also the interconnected cellular networks governing the UPR, mitochondrial function, and ER redox and calcium homeostasis.
