# Corrigendum: Exploring proinsulin proteostasis: insights into beta cell health and diabetes

**DOI:** 10.3389/fmolb.2025.1592000

**Published:** 2025-03-19

**Authors:** Parisima Ghaffarian Zavarzadeh, Kathigna Panchal, Dylan Bishop, Elizabeth Gilbert, Mahi Trivedi, Tovaria Kee, Srivastav Ranganathan, Anoop Arunagiri

**Affiliations:** ^1^ Department of Biological Sciences, East Tennessee State University, Johnson City, TN, United States; ^2^ Max Planck Institute for the Physics of Complex Systems, Dresden, Germany

**Keywords:** proinsulin folding, trafficking, beta cells, proteostasis, insulin biosynthesis, diabetes

In the published article, there was an error in the legend for **Figure 7** as published. A sentence was incomplete in the 7th point in the legend. The corrected legend appears below.

“Orchestrating proinsulin proteostasis in β-cells: Molecular Chaperones, oxidoreductases, and the UPR. (1) PERK ablation, leading to Erp72 upregulation, may impair proinsulin degradation or trafficking. Independently, PERK inhibition can induce ER stress in β-cells, (2) PERK inhibition (by PERKi) prevents eIF2α phosphorylation, negatively impacting the UPR primarily through dysregulation of protein synthesis, (3) Treatment of β-cells with PERKi for 10–12 h induces proinsulin misfolding, (4) BiP inactivation (by SubAB or PERK inhibition) impairs proinsulin folding in β-cells, (5) PDIA6 deletion may impair both the UPR and proinsulin trafficking; Defective proinsulin trafficking could hinder proinsulin folding in the ER, (6) Inhibition of the IRE1α-XBP1 pathway deregulates PDI activity in β-cells, potentially impeding proinsulin folding or disrupting ERAD through impaired PDI-mediated retrotranslocation, (7) Grp170 either directly targets mutant high-molecular weight (HMW) proinsulin aggregates or collaborates with reticulon 3 (RTN3), which engages with PGRMC1 on the ER luminal side (not shown), to clear aggregates via ER-phagy in the cytosol, (8) FBKBP2 knockout results in proinsulin misfolding, (9) High-glucose-induced sulfonylation of PRDX4 impairs its protective function against proinsulin misfolding, (10) Erdj3 may promote proinsulin folding by acting as a co-chaperone with BiP.”

The authors apologize for this error and state that this does not change the scientific conclusions of the article in any way. The original article has been updated.

